# Profiles and Predictors of Dating Violence Among Sexual and Gender Minority Adolescents

**DOI:** 10.1016/j.jadohealth.2020.08.034

**Published:** 2020-10-17

**Authors:** Alexa Martin-Storey, Amanda M. Pollitt, Laura Baams

**Affiliations:** a Department of Psychoeducation, Université de Sherbrooke, Québec, Canada; b Population Research Center, University of Texas at Austin, Austin, Texas; c Department of Pedagogy and Educational Sciences, University of Groningen, Groningen, The Netherlands

**Keywords:** Sexual minority, Gender minority, Dating violence, Discrimination, Victimization, Childhood maltreatment

## Abstract

**Purpose:**

Sexual and gender minority adolescents report higher levels of dating violence compared with their heterosexual and cisgender peers. The objectives of the present study were to (1) identify latent profiles of dating violence; (2) examine if sexual and gender minority adolescents were particularly vulnerable to certain profiles of dating violence; and (3) explore how experiences of peer victimization, discrimination, and parental maltreatment explained this greater vulnerability.

**Methods:**

High school students in Grades 9 and 11 from the 2016 Minnesota Student Survey (N = 87,532; mean age = 15.29 years, SD = 1.23) were asked about their sexual and gender identities, their gender nonconformity, their experiences of verbal, physical, and sexual dating violence victimization and perpetration, as well their experiences of childhood maltreatment, peer victimization, and gender-based and sexual minority status–based discrimination.

**Results:**

Multinomial logistic regression analysis in a three-step latent class analysis procedure suggested five profiles of dating violence victimization and perpetration across the entire sample. Sexual and gender minority adolescents were generally more likely to be in classes high in dating violence victimization, perpetration, or both, compared with their heterosexual and cisgender peers. Gender nonconformity was also associated with greater risk for being in high dating violence classes. These differences, however, were generally nonsignificant when the social stressors of childhood maltreatment, peer victimization, and experiences of discrimination were accounted for.

**Conclusions:**

Although findings suggested greater vulnerability for dating violence among sexual and gender minority adolescents, they underscore the importance of how minority stressors generally accounted for this greater vulnerability for dating violence.

Dating violence (DV) is a common problem among adolescents [1,2] and has serious consequences for physical and mental health, including substance use, suicidality, and depression [3,4]. Patterns of DV victimization and perpetration, however, are more complex than simply some youth are victims and some youth are perpetrators; furthermore, considerable overlap is observed between physical, verbal, and sexual forms of DV. This complexity has implications for health outcomes. Person-centered methodological approaches can illuminate the underlying patterns of victimization and perpetration among adolescents and the relationship dynamics under which different types of DV occur [5–9]. Previous studies with person-centered approaches typically identify three to five DV profiles among adolescents [5–9]. All studies identify a large group of youth who report low levels of DV victimization and perpetration, as well as a smaller group of youth who report high levels of victimization and perpetration over all or most forms of DV. In cases in which multiple types of DV victimization and perpetration (i.e., physical, verbal, and sexual) are accounted for, previous research has generally identified five classes [6,8,10]. These additional classes have included youth who are high on psychological/verbal violence but not the other types of DV [5,6,8,9,11], youth who report higher levels of victimization but not perpetration [7,8,11], youth who are high in perpetration but not victimization [8], and/or youth that report primarily sexual violence but not other types of DV [6,11].

Adolescents who are victimized by peers or family members or who witness intimate partner violence within the family report more DV victimization and perpetration [12–14] likely because these experiences hinder an adolescent’s capacity to develop the skills required to form and maintain healthy relationships. These predictors of DV differ based on the pattern of DV victimization and perpetration and across types of violence [5,6,8,9,11].

Heightened vulnerability to DV exists among youth with sexual minority (i.e., youth with same-sex attraction, same-sex sexual behavior, and/or nonheterosexual identities) and gender minority statuses (e.g., youth whose gender identity differs from their sex as assigned at birth) [1,15,16]. The minority stress model explains health disparities among sexual and gender minority populations as a result of the stigma and subsequent social stress [17]. Indeed, the well-documented higher rates of peer victimization [18,19], parental rejection [20,21], and childhood maltreatment [22,23] are likely central for understanding greater vulnerability for DV among sexual minority and gender diverse youth [24–26]. If variation in exposure to social stressors is associated with different DV profiles, then sexual and gender minority youth, who experience higher rates of these social stressors compared with heterosexual and cisgender youth, may be anticipated to vary in their profiles of DV compared with their peers.

To date, much of the research on DV has combined samples across sexual and gender minority identities, providing an incomplete picture of the tremendous variability within sexual and gender minority populations and the unique risks and vulnerabilities for DV among particular subgroups. Bisexual youth (i.e., youth who report identities indicative of attraction toward individuals of multiple genders) are generally at greater risk for DV than other youth [24,27]. Gender minority youth may also be at increased risk for experiencing DV, starting in adolescence [15]. Transgender youth report higher levels of many of the minority stress factors (i.e., peer victimization and parental rejection) associated with increased risk for DV when compared with sexual minority youth [19,28,29]. Similarly, youth who are gender expansive (i.e., have gender expressions, presentations, or behaviors inconsistent with their sex assigned at birth) are more likely to experience minority stressors such as bias-based peer and family victimization than gender conforming youth [29] and thus may be at particular risk for DV.

## Present Study

Despite differences in the serious consequences for individual health and well-being across patterns of DV, as well as a growing literature examining latent classes of DV among cisgender heterosexual youth, no research to date has examined how sexual and gender minority statuses are associated with profiles of DV. Understanding underlying patterns of DV and differences in these patterns, among sexual and gender minority youth, is essential for determining if they may benefit from existing intervention approaches. Moreover, examining victimization from peers and family, and particularly bias-based victimization, is essential for identifying the mechanisms that render sexual and gender minority youth more vulnerable to DV.

The goals of the present study were to (1) identify patterns of DV victimization and perpetration across different forms of DV; (2) determine how sexual and gender minority status, as well as gender nonconformity more generally, are associated with profiles of DV; and (3) examine to what extent these associations are explained by experiences of peer victimization, discrimination, and childhood maltreatment. We anticipated identifying five classes, including a low involvement class, a high victimization and perpetration class, and a high verbal victimization and perpetration class. We expected that sexual and gender minority statuses would increase the likelihood of membership in the DV victimization and perpetration class, and we anticipated that this vulnerability would be explained by peer victimization, discrimination, and childhood maltreatment.

## Methods

### Study design and sample

The present study uses data from the 2016 Minnesota Student Survey (MSS). The MSS is administered to fifth, eighth, ninth, and 11th grade students every 3 years via local public school districts (or alternative education programs) in Minnesota and managed by the MSS Interagency Team 2016 [30]. Of the 330 operating public school districts that were invited to participate in the survey, 282 (85.5%) participated. Parents of students were asked for passive consent. Students consented to participate, and participation was voluntary and anonymous. The University of Groningen, Department of Pedagogy and Educational Sciences’ Ethics Committee has deemed this study of secondary data to be exempt.

Sexual orientation, gender identity, and gender nonconformity were only assessed in Grades 9 and 11; thus, we excluded Grades 5 and 8 from the current data analyses. In total, the sample comprised 87,532 adolescents enrolled in 348 schools (mean age = 15.29 years, SD = 1.23). Descriptive statistics are presented in Table 1.

### Measures

#### DV victimization and perpetration

Three questions assessed lifetime experiences with verbal, physical, and sexual DV victimization: “Have you ever had a boyfriend or girlfriend in a dating or serious relationship who.” (1) “called you names or put you down verbally?” (2) “hit, slapped or physically hurt you on purpose?” and (3) “pressured you into having sex when you did not want to?” with answer options “Yes” (1) and “No” (0). Three questions asked about perpetration of verbal, physical, and sexual DV, “Have YOU ever done any of the following to a boyfriend or girlfriend in a dating or serious relationship.” (1) “called him/her names or put him/her down verbally?” (2) “hit, slapped or physically hurt him/her on purpose?” (3) “pressured him/her into having sex when he/she did not want to?” with answer options “Yes” (1) and “No” (0). These six items were used as separate indicators in our analyses.

#### Sexual identity, gender identity, and gender nonconformity

One item was used to assess sexual orientation: “Which of the following best describes you?” with answer options “Heterosexual (straight)” (89.6%), “Bisexual” (4.98%), “Gay or Lesbian” (1.27%), and “Not sure (questioning)” (4.06%). One item assessed whether adolescents identified as transgender or another gender minority identity: “Do you consider yourself transgender, genderqueer, genderfluid, or unsure about your gender identity?” with answer options “Yes” (1: 2.68%) and “No” (0) [25]. Gender nonconformity was assessed with one item: “A person’s appearance, style, dress, or the way they walk or talk may affect how people describe them. How do you think other people at school would describe you?” Answer options ranged from 1 to 5 (response options: very or mostly feminine, somewhat feminine, equally feminine and masculine, somewhat masculine, or very or mostly masculine) [31]. Scores were recoded for males so that higher scores indicated higher levels of gender nonconformity.

#### Peer victimization and bias-based bullying

Five items assessed peer victimization, including physical violence, threats of physical violence, spreading rumors, sexual comments, and exclusion from friends and activities [32]. A sample item of this scale is “During the last 30 days, how many times have other students at school pushed, shoved, slapped, hit or kicked you when they were not kidding around?” Answer options ranged from “Never” (1) to “Every day” (5). Items were averaged to create a continuous peer victimization score. To assess bias-based bullying, three continuous items were included and used separately: “During the last 30 days, how often have other students harassed or bullied you for any of the following reasons?” (1) “your gender (being male, female, transgender, etc.),” (2) “your gender expression (your style, dress, or the way you walk or talk),” and (3) “because you are gay, lesbian, or bisexual or because someone thought you were.” Answer options ranged from “Never” (1) to “Every day” (5).

#### Childhood maltreatment

Experiences with parental abuse were assessed with four items and included separately. These included psychological abuse: “Does a parent or other adult in your home regularly swear at you, insult you or put you down?”; physical abuse: “Has a parent or other adult in your household ever hit, beat, kicked or physically hurt you in any way?”; witnessed domestic abuse: “Have your parents or other adults in your home ever slapped, hit, kicked, punched or beat each other up?”; and sexual abuse by a family member: “Has any older or stronger member of your family ever touched you or had you touch them sexually?” with answer options “Yes” (1) and “No” (0).

#### Covariates

Biological sex was assessed with the item “What is your biological sex?” with answer options “Male” (1: 49.43%) and “Female” (0: 50.57%). Age was included as a continuous covariate. Three items assessed ethnicity: all adolescents were asked (yes or no) whether they were Hispanic or Latino/a (9.01%), Somali (1.72%), or Hmong (2.78%). Race was assessed with one item, and adolescents could choose multiple answers: “In addition, what is your race? (if more than one describes you, mark all that apply),” with answer options American Indian or Alaskan Native (5.95%); Asian American (7.97%); black, African, or African American (9.29%); Native Hawaiian or Other Pacific Islander (1.11%); or white (84.72%: reference). For socioeconomic status, one item was included: “Do you currently get free or reduced-price lunch at school?” with answer options “Yes” (27.77%: 1) and “No” (72.23%: 0).

### Analysis plan

To assess patterns of DV victimization and perpetration, latent class analyses (LCAs) were conducted in Mplus version 8.3 [33] and evaluated using two criteria. The first were theoretical; based on extant literature, we anticipated that we would identify five classes. Second, we used four fit statistics to assess model fit: (1) Entropy indicates how well individuals could be classified; larger values indicate a clearer delineation of profiles; (2) the Bayesian Information Criterion indicates model fit; lower values indicate a better model fit; (3) the Vuong–Lo–Mendell–Rubin likelihood ratio; and (4) the Lo–Mendell–Rubin adjusted likelihood ratio test indicate whether a solution with k-classes provided a better model fit than a solution with k – 1 classes.

Full information likelihood was used to account for missingness. A three-step LCA procedure assessed whether sexual and gender minority adolescents were more likely to be classified into different classes; we also tested the association between gender nonconformity and DV classes. The LCA was calculated first. This LCA provides us with the most likely class memberships and accounts for measurement error. Then, the covariates (first sexual and gender minority status and gender nonconformity and then these variables along with the simultaneous inclusion of all social stressor variables) are used to predict the most likely class membership, again accounting for measurement error [34].

We used model constraint in Mplus to assess whether the indirect relations through peer victimization, bias-based bullying, and parental abuse were significant, by calculating the indirect effect of sexual orientation, gender identity, and gender nonconformity on classification into the DV classes for each explanatory factor separately.

## Results

**Figure 1:**
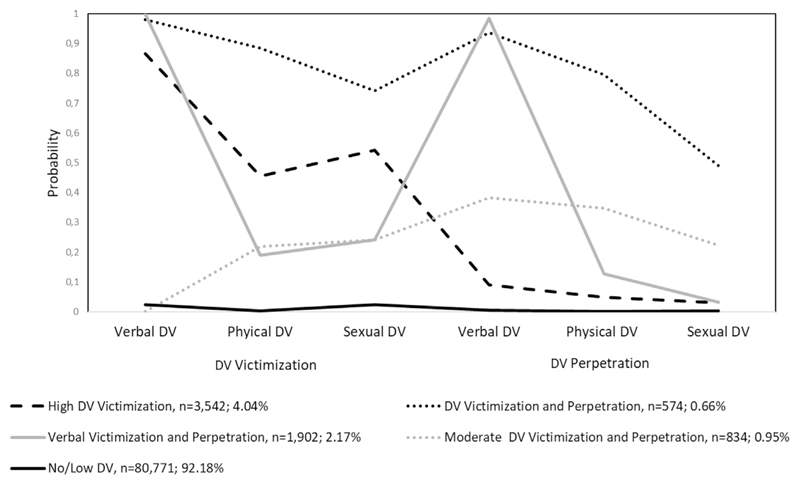
Predicted probabilities of dating violence across five classes. DV, dating violence.

Based on fit statistics from the LCA, we evaluated that the five-class model was the best fitting model for the data (Table 2). For the five-class model, entropy was high (.91), and Bayesian Information Criterion was relatively low, indicating high separation between classes and good fit to the data, respectively. Figure 1 presents probabilities of experiencing each form of DV for each class; probabilities are also presented in Table S1. The first class (no/low DV) consists of participants with relatively low probabilities of experiencing all forms of DV victimization and perpetration. The second class (high DV victimization) consists of participants with relatively high probabilities of experiencing all forms of DV victimization but relatively low probabilities of perpetrating DV. The third class (DV victimization and perpetration) consists of participants with relatively high probabilities of experiencing and perpetrating all forms of DV. The fourth class (verbal victimization and perpetration) consists of participants with relatively high probabilities of experiencing verbal DV victimization and perpetration but lower probabilities of experiencing or perpetrating other forms of DV. The last class (moderate DV victimization and perpetration) consists of participants with moderate probabilities of experiencing and perpetrating DV.

The results of the multinomial logistic regression analysis (Table 3) showed that compared with heterosexual adolescents, gay and lesbian adolescents were more likely to be classified in the high DV victimization and high DV victim and perpetration classes than in the no/low DV class, bisexual adolescents were more likely to be classified in each DV class than in the no/low DV class, and questioning adolescents were more likely to be classified in the high DV victimization class than in the no/low DV class. Compared with nontransgender adolescents, transgender adolescents were more likely to be classified in the high DV victimization and DV victimization and perpetration classes. Finally, higher levels of gender nonconformity were associated with a higher likelihood of being classified in the high DV victimization class, the DV victimization and perpetration, and moderate DV victimization and perpetration classes, compared with the no/low class.

Accounting for peer victimization, sexual minority status and gender-based discrimination, and childhood maltreatment (Table 4), bisexual, gay, and lesbian adolescents, as well as gender minority adolescents, were only significantly different from heterosexual or nontransgender adolescents in their greater likelihood of being in the high victimization, low perpetration class. The indirect relations (Table S2) showed that experiences with peer victimization and bullying based on gender, gender expression, and sexual orientation partially explained every association between sexual orientation, gender identity, and gender nonconformity with classification into each of the DV classes versus the no/low DV class (indirect relations *Ps* < .01).

## Discussion

Understanding patterns of DV among sexual and gender minority adolescents is important because these adolescents are more likely to experience the social stressors that render individuals more vulnerable to DV [18,21,22] and because they are more likely to experience intimate partner violence across the lifespan [27]. In line with some previous research identifying latent classes of DV, the present study identified five profiles of DV victimization and perpetration across the sample [6,8]. These classes were broadly similar to those found in previous studies, including a sizable low victimization–perpetration class (the majority of adolescents) [5–8,11], a class characterized by higher levels of victimization but not perpetration [7,8,11], a class high on all forms of victimization and perpetration [5–8,11], a class high on verbal victimization and perpetration [5,6,8,11], and a class with moderate levels of victimization and perpetration [7,8]. These heterogeneous patterns of DV suggest the importance of validating existing evidence-based universal education approaches to DV programs across different profiles of DV, as this heterogeneity may explain variable success observed with existing programs [35].

Expanding on this literature, compared with heterosexual and nontransgender adolescents, sexual and gender minority adolescents were, in general, more likely to be in classes characterized by higher levels of different patterns of DV. In line with a minority stress framework, which focuses on the role of discrimination and maltreatment from others as underlying factors for vulnerability to negative outcomes among sexual and gender minority populations, the likelihood of being in all the violence classes besides the victimization class was mitigated by victimization, discrimination, and child maltreatment [17]. These findings underscore the relevance of framing the well-documented vulnerability for DV among sexual and gender minority youth [15,24,27] within a minority stress framework [17,27]. Of particular interest is how the inclusion of these stressors explained sexual and gender minority adolescents’ greater likelihood of being in classes that included higher levels of DV perpetration. This unique finding may reflect the role of victimization in aggressive behavior among sexual minority adolescents [36]. More specifically, these findings suggest that prevention and intervention approaches aimed at reducing stigmatizing experiences and preventing peer victimization may also reduce the likelihood of DV among sexual minority adolescents.

Variation in vulnerability to DV was observed across specific sexual minority identities. Supporting previous findings, bisexual adolescents were more likely to be in all the victimization/perpetration classes compared with heterosexual youth [16,27]. This vulnerability likely reflects both how bisexual youth experience discrimination both within and outside of sexual minority communities [37], as well as the more intense sexualization (i.e., a process whereby individuals are evaluated primarily by their sexual value to others) of bisexual youth that may place them at elevated risk for certain kinds of DV, such as sexual violence. The particular vulnerability of bisexual youth across almost all profiles of sexual violence suggests that intervention and prevention approaches need to address the needs of these youth specifically.

Gender identity was associated with vulnerability for patterns of DV. Supporting a minority stress framework [19], transgender adolescents were more likely to be in the high victimization and perpetration group, but this vulnerability was explained via discrimination, peer victimization, and childhood maltreatment. A suppressor effect was observed whereby transgender adolescents were more likely to be in the high victimization, low perpetration group after accounting for the social stressors. Although this finding needs to be replicated, it further highlights the role of multiple forms of victimization for outcomes among transgender youth [29].

Gender nonconformity increased the likelihood of youth being in most DV victimization and perpetration classes, differences that persisted for the high victimization, low perpetration class and the moderate victimization and perpetration class after controlling for social stressors. These findings may reflect a literature linking distress regarding failure to conform to gender roles among boys to DV perpetration specifically [38] and higher endorsement of masculinity to aggressive behavior among cis-gender girls and women more generally [39,40].

Furthermore, bullying based on gender and gender expression, but not sexual orientation, predicted higher likelihood of being in the DV classes for transgender and gender-expansive adolescents. The present study asked participants to describe the extent to which others felt they conformed to gender roles and not how they themselves felt about their gender expression. Because of the formulation of this question, discrimination because of gender expression and participant’s perceptions of how others perceived their gender presentation may overlap. Despite these limitations, this is the first study to examine how concurrent gender nonconformity is associated with patterns of DV victimization and perpetration. Together, these findings suggest that the extent to which youth conform to gender norms, whether via their identities or via other aspects of their appearance and behavior, may play an outsized role in vulnerability to DV. Considering that DV—whether it occurs through perpetration or victimization—is a gendered form of violence [41], it is not surprising that this visible and stigmatized characteristic places youth at risk. Our results indicate that addressing discrimination and stigma based on gender, gender identity, and gender expression is critical for the safety of sexual and gender minority youth.

### Limitations and future directions

The present study used an increasingly popular method, LCA, for assessing heterogeneity in DV to nuance the understanding of DV among sexual and gender minority youth. Despite these strengths, these findings should be interpreted in light of several limitations. First, as would be expected for a survey of this size, single item questions were used to assess each type of DV victimization and perpetration, and the response option was dichotomous, limiting our capacity to assess revictimization. Second, because of the demographic makeup of the state of Minnesota, examining the experiences of sexual and gender minority youth at the intersection of race/ethnicity was not possible. Future research that targets specific subpopulations should address this limitation. Third, future research should include additional options for sexual identity (such as queer), as well as more gender-neutral language regarding dating partners. Finally, these data were correlational and cross-sectional, and directionality regarding the associations observed cannot be inferred.

Because of the serious consequences of DV [4], the vulnerability for DV among sexual and gender minority youth is receiving increasing levels of interest [35]. Ultimately, these findings suggest the importance of future work examining how DV functions as a minority stressor in understanding psychosocial vulnerability among sexual and gender minority youth.

## Supplementary Material

Supplementary data related to this article can be found at https://doi.org/10.1016/j.jadohealth.2020.08.034.

Supplementary Material

## Figures and Tables

**Table 1:** Descriptive statistics key variables across sexual orientation and gender identity groups

	Lesbian/gay (N = 4,014)	Bisexual (N = 3,272)	Questioning (N = 2,168)	Transgender (N = 78,761)	Nontransgender (N = 72,305)	Heterosexual (N = 1,027)
DV *% occurrence*						
Verbal DV victimization, % (n)	20.78 (198)	24.78 (944)	11.64 (347)	21.33 (421)	9.42 (6,996)	8.65 (5,900)
Physical DV victimization, % (n)	11.31 (108)	12.03 (458)	5.72 (170)	10.55 (208)	4.11 (3,053)	3.69 (2,518)
Sexual DV victimization, % (n)	13.64 (130)	19.39 (737)	9.15 (272)	16.67 (328)	6.23 (4,622)	5.58 (3,801)
Verbal DV perpetration, % (n)	8.41 (80)	8.55 (325)	5.07 (150)	7.41 (146)	4.40 (3,258)	4.17 (2,838)
Physical DV perpetration, % (n)	3.46 (33)	3.92 (149)	2.54 (75)	3.96 (78)	1.64 (1,217)	1.52 (1,037)
Sexual DV perpetration, % (n)	3.89 (37)	1.61 (61)	2.17 (64)	3.76 (74)	1.11 (822)	1.07 (731)
Peer victimization, mean (SD)	1.45 (.68)	1.51 (.68)	1.35 (.60)	1.57 (.77)	1.24 (.46)	1.23 (.44)
Bullying *% every day,* % (n)						
Bullying based on gender	3.21 (32)	1.27 (50)	1.50 (47)	4.70 (98)	.31 (241)	.29 (207)
Bullying based on gender expression	4.31 (43)	2.48 (98)	2.64 (83)	5.84 (122)	.83 (643)	.75 (535)
Bullying based on sexual orientation	7.02 (70)	2.78 (110)	1.97 (62)	6.75 (141)	.46 (355)	.35 (246)
Parental abuse *% occurrence,* % (n)						
Psychological parental abuse	28.26 (269)	31.81 (1,203)	20.07 (596)	32.09 (630)	12.83 (9,502)	11.81 (8,043)
Physical parental abuse	22.79 (217)	24.51 (926)	17.14 (509)	24.48 (482)	11.18 (8,264)	10.42 (7,079)
Witnessing domestic abuse	12.67 (121)	14.65 (554)	10.39 (309)	14.61 (288)	6.12 (4,529)	5.62 (3,816)
Sexual abuse by family member	8.73 (83)	8.65 (325)	5.00 (148)	9.23 (182)	2.11 (1,588)	1.74 (1,183)

Sample sizes of sexual and gender identity groups differ for specific measures. DV = dating violence.

**Table 2:** Fit statistics for complex three-step latent class analyses on dating violence victimization and perpetration (N = 87,532)

	Entropy	BIC	Vuong–Lo– Mendell–Rubin LRT	Lo–Mendell– Rubin adjusted LRT
Two classes	.908	141,770	<.001	<.001
Three classes	.925	140,416	<.001	<.001
Four classes	.932	140,012	<.001	<.001
Five classes	.909	139,653	<.001	<.001
Six classes	.826	139,575	.002	.002
Seven classes	.839	139,521	<.001	<.001

Entropy refers to how well individual cases can be classified into classes; larger values indicate distinctive classes. Bayesian Information Criterion (BIC) is a measure of model fit; lower values indicate that the estimated model is more likely to be the true model. Vuong–Lo–Mendell-Rubin likelihood ratio test (LRT) and the Lo–Mendell–Rubin adjusted LRT indicate whether a solution with k-classes provides a better fit to the data than a solution with k – 1 classes; a nonsignificant *p* value (*p* > .05) indicates that a solution with one more class is not needed.

**Table 3:** The multinomial regression model of dating violence classes predicted by sexual orientation, gender identity, and gender nonconformity (N = 71,843)

	High DV victimization versus no/low DV			DV victimization and perpetration versus no/low DV			Verbal victimization and perpetration versus no/low DV			Moderate DV victimization and perpetration versus no/low DV		
	Estimate (SE)	RRR	*p*	Estimate (SE)	RRR	*p*	Estimate (SE)	RRR	*p*	Estimate (SE)	RRR	*p*
Sexual orientation (ref: heterosexual)												
Gay or lesbian	**–.95 (.16)**	**2.59**	**< .001**	**–.94 (.31)**	**2.56**	**.002**	–.24 (.29)	1.27	.410	–.41 (.32)	1.51	.194
Bisexual	**–1.36 (.07)**	**3.90**	**< .001**	**–.65 (.24)**	**1.92**	**.007**	**–.43 (.15)**	**1.54**	**.003**	**–.63 (.18)**	**1.87**	**.001**
Questioning	**–.42 (.11)**	**1.53**	**< .001**	–.39 (.27)	1.47	.156	.09 (.17)	.92	.613	–.21 (.22)	1.23	.338
Transgender (ref: nontransgender)	–.00 (.10)	1.00	.983	**–.73 (.25)**	**2.08**	**.004**	–.01 (.23)	1.01	.982	–.22 (.24)	1.25	.362
Gender nonconformity	**– .26 (.03)**	**1.30**	**< .001**	**–.21 (.07)**	**1.23**	**.003**	–.08 (.04)	1.08	.054	**– .18 (.05)**	**1.20**	**< .001**
Biological sex (ref: female)	**1.05 (.06)**	**.35**	**< .001**	–.07 (.12)	1.07	.567	**.79 (.07)**	**.46**	**< .001**	**.19 (.09)**	**.83**	**.031**

Controlling for age and race/ethnicity. Unstandardized estimates and relative risk ratios (RRR). Statistically significant estimates (p < .05) are shown in bold. DV = dating violence.

**Table 4:** Multinomial regression models of dating violence classes predicted by sexual orientation, gender identity, and gender nonconformity, and including peer victimization, bias-based bullying, and parental abuse (N = 70,071)

	High DV victimization versus no/low DV			DV victimization and perpetration versus no/low DV			Verbal victimization and perpetration versus no/low DV			Moderate DV victimization and perpetration versus no/ low DV		
	Estimate (SE)	RRR	*p*	Estimate (SE)	RRR	*p*	Estimate (SE)	RRR	*p*	Estimate (SE)	RRR	*p*
Sexual orientation (ref: heterosexual)												
Gay or lesbian	**–.62 (.19)**	**1.86**	**.001**	–.19 (.36)	1.21	.601	–.15 (.28)	1.16	.595	.03 (.37)	.97	.934
Bisexual	**–.98 (.08)**	**2.67**	**< .001**	–.15 (.28)	1.16	.584	–.15 (.15)	1.16	.313	–.24 (.22)	1.27	.272
Questioning	–.18 (.12)	1.20	.115	.42 (.34)	.66	.216	.24 (.17)	.78	.160	.11 (.24)	.89	.637
Transgender (ref: nontransgender)	**.29 (.13)**	**.75**	**.027**	.00 (.32)	1.00	.991	.19 (.22)	.83	.391	.11 (.28)	.90	.691
Gender nonconformity	**–.11 (.03)**	**1.12**	**< .001**	–.05 (.07)	1.05	.476	.00 (.04)	1.00	.939	**–.11 (.05)**	**1.12**	**.037**
Peer victimization	**–1.04 (.06)**	**2.83**	**< .001**	**–1.13 (.09)**	**3.09**	**< .001**	**– .58 (.08)**	**1.79**	**< .001**	**– .87 (.10)**	**2.38**	**< .001**
Bullying based on gender	**–.12 (.06)**	**1.12**	**.039**	**–.34 (.10)**	**1.40**	**.001**	**–.18 (.06)**	**1.20**	**.004**	**–.19 (.09)**	**1.20**	**.041**
Bullying based on gender expression	**– .32 (.04)**	**1.38**	**< .001**	–.02 (.09)	1.02	.794	**– .37 (.05)**	**1.45**	**< .001**	**– .22 (.07)**	**1.25**	**.001**
Bullying based on sexual orientation	–.06 (.06)	1.06	.315	–.03 (.09)	1.03	.753	.12 (.08)	.88	.103	–.13 (.09)	1.13	.170
Psychological parental abuse	**–.72 (.07)**	**2.05**	**< .001**	**–.57 (.17)**	**1.77**	**.001**	**– .62 (.09)**	**1.86**	**< .001**	**– .73 (.13)**	**2.07**	**< .001**
Physical parental abuse	**–.48 (.08)**	**1.62**	**< .001**	**–.60 (.19)**	**1.82**	**.001**	**– .52 (.09)**	**1.69**	**< .001**	–.25 (.14)	1.29	.079
Witnessing domestic abuse	**–.61 (.09)**	**1.84**	**< .001**	**–1.69 (.18)**	**5.41**	**< .001**	**–.49 (.11)**	**1.63**	**< .001**	**–.99 (.13)**	**2.69**	**< .001**
Sexual abuse by family member	**–1.13 (.11)**	**3.08**	**< .001**	**–2.52 (.18)**	**12.48**	**< .001**	**–.97 (.15)**	**2.65**	**< .001**	**– 1.26 (.19)**	**3.51**	**< .001**

Controlling for biological sex, age, and race/ethnicity. Unstandardized estimates and relative risk ratios (RRR). Statistically significant estimates (p < .05) are shown in bold.

DV = dating violence; SE = standard error.
