# Supplementary material for: Profiles and Predictors of Dating Violence Among Sexual and Gender Minority Adolescents
Source: J Adolesc Health. Author manuscript; Available in PMC 2022 Jun 4. (PMC7612810; doi:10.1016/j.jadohealth.2020.08.034)
Supplement: Supplementary Material [file EMS145487-supplement-Supplementary_Material.docx]

Supplemental Table 1

*Probabilities of Experiences with Forms of Dating Violence Across 5 Latent Classes*

|  |  | No/low DV  (92.18) | High DV Victimization  (4.04%) | DV Victimization and Perpetration  (0.66%) | Verbal Victimization and Perpetration  (2.17%) | Moderate DV Victimization and Perpetration  (0.95%) |
| --- | --- | --- | --- | --- | --- | --- |
| Victimization | Verbal DV | 0.023 | 0.867 | 0.981 | 1.000 | 0.000 |
|  | Phyical DV | 0.004 | 0.455 | 0.885 | 0.190 | 0.218 |
|  | Sexual DV | 0.023 | 0.542 | 0.741 | 0.242 | 0.241 |
| Perpetration | Verbal DV | 0.006 | 0.090 | 0.937 | 0.985 | 0.382 |
|  | Physical DV | 0.000 | 0.049 | 0.795 | 0.127 | 0.347 |
|  | Sexual DV | 0.002 | 0.030 | 0.490 | 0.033 | 0.222 |

DV: Dating violence

Supplemental Table S2

*Indirect Effects from Sexual Orientation and Gender Identity/Nonconformity to DV Classes, Via Peer and Parent Experiences (N = 69,420)*

|  | High DV Victimization vs No/low DV | | DV Victimization and Perp vs No/low DV | | Verbal Victimization and Perpetration vs No/low DV | | Moderate DV Victimization and Perpetration vs No/low DV | |
| --- | --- | --- | --- | --- | --- | --- | --- | --- |
|  | Indirect through peer victimization | | | | | | | |
|  | Estimate (SE) | p | Estimate (SE) | p | Estimate (SE) | p | Estimate (SE) | p |
| Sexual orientation (ref: heterosexual) |  |  |  |  |  |  |  |  |
| Gay or lesbian | .11 (.03) | < .001 | .11 (.03) | < .001 | .09 (.02) | < .001 | .09 (.02) | < .001 |
| Bisexual | .24 (.02) | < .001 | .24 (.02) | < .001 | .20 (.02) | < .001 | .20 (.02) | < .001 |
| Questioning | .09 (.02) | < .001 | .10 (.02) | < .001 | .08 (.01) | < .001 | .08 (.01) | < .001 |
| Transgender (ref: non-transgender) | .18 (.02) | < .001 | .19 (.03) | < .001 | .15 (.02) | < .001 | .15 (.02) | < .001 |
| Gender nonconformity | .05 (.00) | < .001 | .06 (.00) | < .001 | .05 (.00) | < .001 | .05 (.00) | < .001 |
|  | Indirect through bullying based on gender | | | | | | | |
| Sexual orientation (ref: heterosexual) |  |  |  |  |  |  |  |  |
| Gay or lesbian | .11 (.02) | < .001 | .13 (.02) | < .001 | .10 (.02) | < .001 | .10 (.02) | < .001 |
| Bisexual | .07 (.01) | < .001 | .08 (.01) | < .001 | .06 (.01) | < .001 | .06 (.01) | < .001 |
| Questioning | .05 (.01) | < .001 | .06 (.01) | < .001 | .04 (.01) | < .001 | .04 (.01) | < .001 |
| Transgender (ref: non-transgender) | .25 (.02) | < .001 | .30 (.03) | < .001 | .22 (.02) | < .001 | .22 (.03) | < .001 |
| Gender nonconformity | .02 (.00) | < .001 | .02 (.00) | < .001 | .02 (.00) | < .001 | .02 (.00) | < .001 |
|  | Indirect through bullying based on gender expression | | | | | | | |
| Sexual orientation (ref: heterosexual) |  |  |  |  |  |  |  |  |
| Gay or lesbian | .18 (.02) | < .001 | .17 (.03) | < .001 | .16 (.02) | < .001 | .14 (.02) | < .001 |
| Bisexual | .15 (.01) | < .001 | .14 (.01) | < .001 | .13 (.01) | < .001 | .12 (.01) | < .001 |
| Questioning | .08 (.01) | < .001 | .07 (.01) | < .001 | .07 (.01) | < .001 | .06 (.01) | < .001 |
| Transgender (ref: non-transgender) | .21 (.02) | < .001 | .20 (.02) | < .001 | .19 (.02) | < .001 | .17 (.02) | < .001 |
| Gender nonconformity | .06 (.00) | < .001 | .05 (.00) | < .001 | .05 (.00) | < .001 | .04 (.00) | < .001 |
|  | Indirect through bullying based on sexual orientation | | | | | | | |
| Sexual orientation (ref: heterosexual) |  |  |  |  |  |  |  |  |
| Gay or lesbian | .41 (.03) | < .001 | .46 (.05) | < .001 | .26 (.04) | < .001 | .38 (.05) | < .001 |
| Bisexual | .23 (.02) | < .001 | .25 (.03) | < .001 | .15 (.02) | < .001 | .21 (.03) | < .001 |
| Questioning | .08 (.01) | < .001 | .09 (.01) | < .001 | .05 (.01) | < .001 | .07 (.01) | < .001 |
| Transgender (ref: non-transgender) | .20 (.02) | < .001 | .23 (.03) | < .001 | .13 (.02) | < .001 | .19 (.03) | < .001 |
| Gender nonconformity | .02 (.00) | < .001 | .03 (.00) | < .001 | .02 (.00) | < .001 | .02 (.00) | < .001 |
|  | Indirect through psychological parental abuse | | | | | | | |
| Sexual orientation (ref: heterosexual) |  |  |  |  |  |  |  |  |
| Gay or lesbian | .12 (.02) | < .001 | .16 (.03) | < .001 | .10 (.02) | < .001 | .11 (.02) | < .001 |
| Bisexual | .17 (.01) | < .001 | .23 (.02) | < .001 | .15 (.01) | < .001 | .16 (.02) | < .001 |
| Questioning | .06 (.01) | < .001 | .08 (.02) | < .001 | .05 (.01) | < .001 | .05 (.01) | < .001 |
| Transgender (ref: non-transgender) | .08 (.02) | < .001 | .11 (.02) | < .001 | .07 (.01) | < .001 | .07 (.02) | < .001 |
| Gender nonconformity | .04 (.00) | < .001 | .05 (.00) | < .001 | .05 (.00) | < .001 | .03 (.00) | < .001 |
|  | Indirect through physical parental abuse | | | | | | | |
| Sexual orientation (ref: heterosexual) |  |  |  |  |  |  |  |  |
| Gay or lesbian | .09 (.02) | < .001 | .14 (.03) | < .001 | .08 (.02) | < .001 | .08 (.02) | < .001 |
| Bisexual | .13 (.01) | < .001 | .20 (.02) | < .001 | .12 (.01) | < .001 | .11 (.01) | < .001 |
| Questioning | .05 (.01) | < .001 | .08 (.02) | < .001 | .05 (.01) | < .001 | .04 (.01) | < .001 |
| Transgender (ref: non-transgender) | .04 (.01) | .004 | .06 (.02) | .005 | .03 (.01) | .004 | .03 (.01) | .006 |
| Gender nonconformity | .02 (.00) | < .001 | .03 (.00) | < .001 | .02 (.00) | < .001 | .02 (.00) | < .001 |
|  | Indirect through witnessing domestic abuse | | | | | | | |
| Sexual orientation (ref: heterosexual) |  |  |  |  |  |  |  |  |
| Gay or lesbian | .04 (.01) | .003 | .08 (.03) | .004 | .04 (.01) | .003 | .05 (.02) | .003 |
| Bisexual | .08 (.01) | < .001 | .14 (.02) | < .001 | .07 (.01) | < .001 | .09 (.01) | < .001 |
| Questioning | .03 (.01) | < .001 | .06 (.02) | < .001 | .03 (.01) | < .001 | .04 (.01) | < .001 |
| Transgender (ref: non-transgender) | .03 (.01) | .007 | .06 (.02) | .007 | .03 (.01) | .007 | .04 (.01) | .008 |
| Gender nonconformity | .02 (,00) | < .001 | .03 (.00) | < .001 | .01 (.00) | < .001 | .02 (.01) | < .001 |
|  | Indirect through sexual abuse by family member | | | | | | | |
| Sexual orientation (ref: heterosexual) |  |  |  |  |  |  |  |  |
| Gay or lesbian | .07 (.01) | < .001 | .13 (.03) | < .001 | .06 (.01) | < .001 | .07 (.02) | < .001 |
| Bisexual | .07 (.01) | < .001 | .14 (.02) | < .001 | .07 (.01) | < .001 | .07 (.01) | < .001 |
| Questioning | .03 (.01) | < .001 | .06 (.01) | < .001 | .03 (.01) | < .001 | .03 (.01) | < .001 |
| Transgender (ref: non-transgender) | .04 (.01) | < .001 | .09 (.02) | < .001 | .04 (.01) | < .001 | .05 (.01) | < .001 |
| Gender nonconformity | .01 (.00) | < .001 | .02 (.00) | < .001 | .01 (.00) | < .001 | .01 (.00) | < .001 |

*Note*. Controlling for biological sex, age, and race/ethnicity.
